# Relation of Adiponectin to Glucose Tolerance Status, Adiposity, and Cardiovascular Risk Factor Load

**DOI:** 10.1155/2012/250621

**Published:** 2011-12-27

**Authors:** N. Wolfson, D. Gavish, Z. Matas, M. Boaz, M. Shargorodsky

**Affiliations:** ^1^Department of Medicine, Edith Wolfson Medical Center and Sackler School of Medicine, Tel Aviv University, Tel Aviv 69978, Israel; ^2^Department of Biochemistry, Edith Wolfson Medical Center, Holon 58100, Israel; ^3^Epidemiology and Research Unit, Edith Wolfson Medical Center and Sackler School of Medicine, Tel Aviv University, Tel Aviv 69978, Israel; ^4^Department of Endocrinology, Edith Wolfson Medical Center and Sackler School of Medicine, Tel Aviv University, P. O. Box 5, Holon 58100, Israel

## Abstract

*Objective*. Adiponectin has anti-atherogenic and anti-inflammatory properties. We investigated the influence of adiponectin on glucose tolerance status, adiposity and cardiovascular risk factors (CVRFs). *Design and Patients*. Study consisted of 107 subjects: 55 with normal glucose tolerance (NGT) and 52 with impaired glucose regulation (IGR) who were divided into two groups: 24 subjects with impaired fasting glucose (IFG Group) and 28 patients with type 2 diabetes mellitus (DM Group). In additional analysis, study participants were divided into two groups, according to CVRFs: low and high risk. Measurements: Patients were evaluated for glucose, HbA1C, insulin, lipids, CRP, HOMA-IR and adiponectin. *Measurements*. Patients were evaluated for glucose, HbA1C, insulin, lipids, CRP, HOMA-IR and adiponectin. *Results*. Adiponectin was significantly higher in NGT group than in IFG (*P* = 0.003) and DM (*P* = 0.01) groups. Adiponectin was significantly, positively associated with HDL and inversely associated with glucose, HbA1c, ALT, AST, TG, HOMA-IR. Patients with higher CVRFs load have lesser adiponectin compared to patients with low cardiovascular risk *P* < 0.0001). Adiponectin was inversely associated with the number of risk factors (*r* = −0.430, *P* = 0.0001). *Conclusions*. Circulating adiponectin was significantly lower in subjects with different degree of IGR compared to subjects with normal glucose homeostasis. Adiponectin was significantly lower in high risk group than low risk group and decreased concurrently with increased number of CVRFs.

## 1. Introduction

Adiponectin, a collagen-like protein specifically and highly expressed in human adipose cells, plays an important role in insulin sensitivity, inflammation, lipid metabolism, and atherogenesis [1–3]. Low plasma adiponectin levels are significantly correlated with endothelial dysfunction, increased intima media thickness, and progression of coronary artery calcification independently of other cardiovascular risk factors [4–6]. Plasma adiponectin levels are reduced not only among obese patients but also in disease states frequently associated with obesity, such as type 2 diabetes, hypertension, metabolic syndrome, and coronary artery disease [7–9]. Altogether, these data suggest that adiponectin may mediate its effects via obesity-independent mechanisms, but whether obesity per se or other pathways play a regulatory role is unclear and deserves further evaluation. The present study was designed to assess the relation between plasma adiponectin levels to glucose tolerance status, adiposity, and cardiovascular risk factors (CVRFs) load.

## 2. Methods

The study group consisted of 107 Caucasian subjects, (67 females, mean age 56.4 ± 10.0 years) who were recruited from the outpatient metabolic clinic and evaluated for the study. The study participants were divided into three groups according to glucose tolerance status: 55 with normal glucose tolerance (NGT Group), 24 subjects with impaired fasting glucose (IFG Group), and 28 patients with type 2 diabetes mellitus (DM Group).

The cutoff for IFG was defined as FBG = 100 − 125 mg/dL, according to current ADA criteria [10].

In additional analysis study, participants were divided into two groups, according to CVRFs: low- and high-risk subjects. Cardiovascular risk factors were defined using the ATP III definition and included the following disorders: hypertriglyceridemia (≥150 mg/dL and/or pharmacological treatment), low HDL cholesterol level (<40 mg/dL in men and <50 mg/dL in women), high blood pressure (systolic blood pressure ≥130 mmHg and/or diastolic BP ≥85 mmHg and/or pharmacological treatment), and elevated plasma glucose ≥100 mg/dL and/or pharmacological treatment). Obesity was defined using the World Health Organization criteria (BMI ≥30 kg/m²). According to the above-mentioned CVRFs, the high-risk group included patients with three or more CVRFs (*n* = 55), whereas low risk had two or less CVRFs (*n* = 52). 

### 2.1. Biochemical Parameters

Blood sampling for full chemistry and metabolic parameters, including fasting glucose, HbA1C, c-peptide, insulin, lipids profile, fibrinogen, hs-CRP, and plasma adiponectin, will be performed. Adiponectin will be determined by a commercial sandwich enzyme immunoassay technique, R&D Systems, Minneapolis, MN, USA (catalog number DRP300) with 2.8% intra-assay and 6.5% interassay variability.

Insulin resistance and *β*-cell function was estimated using homeostasis model assessment. Homeostasis model assessment-insulin resistance (HOMA-IR) will be calculated by the following formula: fasting plasma insulin (mU/mL) × fasting plasma glucose (mg/dL)/405 [11], HOMA for *β*-cell function (HOMA-*β*) as follows: 20 × fasting plasma insulin (mU/mL)/fasting plasma glucose (mmol/L)−3.5.

### 2.2. Statistical Analysis

Analysis of data was carried out using SPSS 9.0 statistical analysis software (SPSS Inc., Chicago, IL, USA, 1999). For continuous variables, such as hemodynamic and biochemistry measures, descriptive statistics were calculated and reported as mean ± standard deviation. Distributions of continuous variables were assessed for normality using the Kolmogorov-Smirnov test (cutoff at  *P* = 0.01). Associations between continuous variables with approximately normal distributions including anthropometric, metabolic, and hemodynamic parameters were described using Pearson's correlation coefficients. Associations between continuous variables with distributions significantly deviating from normal were described using Spearman's rho coefficients. All tests are twosided and considered significant at  *P* < 0.05. 

## 3. Results

Demographic and clinical characteristics of the study groups according to glucose tolerance status are presented in Table 1. As can be seen, all groups were similar in terms of age, BMI, and presence of concomitant cardiovascular risk factors such as hypertension, dyslipidemia, and smoking. Fasting glucose differed significantly between each group and every other group, lowest in NGT subjects and highest in patients with DM, as expected. HOMA-IR as well as fasting insulin was significantly lower in NGT group than in IFG and DM groups, but not different between IFG and DM groups. Additionally, significant intergroup differences were detected for HDL-cholesterol and triglycerides.

Adiponectin was significantly higher in NGT group than in IFG (*P* = 0.003) and DM (*P* = 0.01) groups. A difference in plasma adiponectin levels between IFG and DM groups was not detected.

As shown in Table 2, adiponectin was significantly, positively associated with HDL, significantly inversely associated with glucose, HbA1c, ALT, AST, triglycerides, insulin, HOMA-IR, and marginally inversely associated with CRP. No association between adiponectin and BMI was observed (*r* = −0.008, *P* = 0.931). Multiple regression models which were arrived at using a backward, stepwise approach were performed to identify variables independently associated with plasma adiponectin levels. In this model, adiponectin was significantly inversely associated with HbA1C (standardized beta = −0.385, *P* = 0.009) and ALT (standardized beta = −0.453, *P* = 0.040). The overall model is significant (*P* < 0.0001) and explains 38.2% of variability in plasma adiponectin levels.

In additional statistical analysis, the study participants were divided into two groups according to the presence of CVRFs: low-risk and high-risk. As can be seen in Table 3, age and BMI were not different between low-risk and high-risk patients. High risk-subjects had a median of 3 CVRFs (range 3–4), while low-risk subjects had a median of 2 CVRFs (range 0–2). Systolic and diastolic blood pressure as well as fasting glucose, HbA1C, fasting insulin, HOMA-IR, triglycerides, AST, and LDL-cholesterol were significantly higher and HDL-cholesterol significantly lower in high versus low-risk patients. Plasma adiponectin levels were significantly lower in high-risk group compared to low-risk subjects (6755.6 ± 3492.2 versus 13701.1 ± 7051.5 ng/mL, *P* < 0.0001).

Adiponectin was significantly inversely associated with the number of risk factors (*r* = −0.430, *P* = 0.0001) (Table 4). 

## 4. Discussion

In the present study, circulating adiponectin was significantly lower in subjects with different degree of IGR compared to subjects with normal glucose homeostasis. Furthermore we confirm previously observed positive correlations of adiponectin with HDL and negative correlations with glucose, HbA1c, triglycerides, insulin, and HOMA-IR and report an inverse correlation with ALT. In multiple regression models HbA1C as well as ALT was independent determinant of adiponectin levels and explained 38.2% of variability in plasma adiponectin levels. Additionally, adiponectin was significantly lower in high-risk group than low-risk group and decreased concurrently with increased number of CVRFs. Thus, hypoadiponectinemia is more intensively related to glucose intolerance and CVRFs load than to adiposity.

 The site and mechanism of adiponectin actions on glucose metabolism remain unknown. Recent genome-wide scans have mapped a diabetes susceptibility locus to chromosome 3q27, where the adiponectin gene (apM1) is located [12]. Evidence of an association between type 2 diabetes and single nucleotide polymorphisms at positions 45 and 276 and in the proximal promoter and exon 3 of the adiponectin gene has been also reported [11]. Furthermore, it has been shown that circulating adiponectin levels are significantly lower in healthy first-degree relatives of type 2 diabetic patients [13].

In our study, plasma adiponectin levels were significantly lower in both IFG and DM groups compared to subjects with normal glucose tolerance. Moreover, HbA1C levels were independent determinant of circulating adiponectin. Therefore, there is a scientific rationale that plasma adiponectin is a target for future research in the treatment and prevention of diabetes. We suggest that increase in adiponectin levels by combination of lifestyle modifications and medications may have a beneficial effect in patients with impaired glucose regulation.

Our study confirmed previously observed strong inverse associations between low adiponectin levels and some of the well-known risk factors for atherosclerosis, such as low HDL-cholesterol levels, hypertriglyceridemia, and insulin resistance [14–16]. Additionally, we found significant inverse correlation between adiponectin and ALT, a surrogate marker for nonalcoholic fatty liver disease, which has recently been considered as a predictor for development of type 2 diabetes and metabolic syndrome [17–19]. These findings clearly support the point of view that adiponectin may have an active role in the pathogenesis of cardiovascular diseases.

 In the present study, no associations of adiponectin with BMI have been found; however, other studies have been unable to detect associations between adiponectin and whole-body fat mass and subcutaneous fat volume [20]. Nevertheless, an association between BMI and circulating adiponectin levels has been reported by other authors [21, 22]. Although we did not find the associations of adiponectin with BMI as well as differences in adiponectin levels between obese (BMI ≥30 kg/m²) and nonobese study participants, the possibility that the relatively small sample size in the present study limited exploration of these potential correlations cannot be excluded.

 The present study observed strong associations between adiponectin levels and glucose tolerance status as well as number of cardiovascular risk factors. Thus, despite the fact that adiponectin is highly specific to adipose tissue, hypoadiponectinemia is more intensively related to impaired glucose regulation and CVRFs load than to adiposity. The findings of our study are in accordance with published data that circulating adiponectin level is a strong risk marker for metabolic syndrome independent of measures of adiposity [15]. Moreover, decreased plasma adiponectin levels are independently associated with the presence of coronary artery disease in men even after adjustment for BMI [23]. Among healthy men in the Health Professionals Follow-up Study, a doubling of adiponectin levels was associated with a 30% decreased risk for myocardial infarction after adjustment for BMI, alcohol consumption, physical activity, diabetes, and hypertension [24]. Similarly, among patients with type 1 and type 2 diabetes mellitus, increased circulating adiponectin levels were associated with a lower risk of coronary artery disease after adjusting for standard risk factors [25, 26]. These combined findings indicate that adiponectin may have a direct antiatherogenic role or mediate its effects via obesity-independent mechanisms. 

 Thus, plasma adiponectin integrates with the cardiovascular risk factors and is a potential target for research in reducing morbidity and mortality of atherosclerotic disease. Future research should focus on clinical endpoints such as prevention of diabetes and reduction of cardiovascular morbidity and mortality following adiponectin increase, as well as the ability of adiponectin values to predict these outcomes.

## Figures and Tables

**Table 1:** Clinical characteristics of study patients by glucose tolerance status.

Variables	NGT group *n* = 55	IFG group *n* = 24	DM group *n* = 28
Female/male	39/16	7/17	17/11
Age (y)	55.7 ± 9.5	58.8 ± 9.6	55.9 ± 11.3
BMI (kg/m²)	33.3 ± 6.5	32.9 ± 6.8	31.6 ± 6.2
Current smokers, *n* (%)	7 (13%)	3 (8%)	3 (11%)
Hypertension, *n* (%)	28 (52%)	10 (42%)	15 (54%)
Dyslipidemia, *n* (%)	24 (44%)	12 (50%)	17 (61%)
Systolic blood pressure (mmHg)	131.5 ± 18.4	134.7 ± 18.7	134.9 ± 19.6
Diastolic blood pressure (mmHg)	73.4 ± 14.1	76.5 ± 12.1	76.0 ± 9.6
Fasting glucose (mg/dL)	89.5 ± 7.3	109.4 ± 7.6*	156.0 ± 53.5*
HBAIC (%)	5.7 ± 0.4	5.9 ± 0.5*	8.0 ± 2.1*
HDL-cholesterol (mg/dL)	56.6 ± 21.3	47.6 ± 13.4*	45.9 ± 18.3*
Triglycerides (mg/dL)	119.9 ± 59.4	150.6 ± 62.0*	195.3 ± 158.4*
Hs-CRP (mg/dL)	0.9 ± 0.4	0.7 ± 1.2	0.9 ± 1.1
ALT (mg/dL)	26.2 ± 12.8	30.9 ± 16.2	33.6 ± 29.1*
AST (mg/dL)	22.9 ± 7.8	27.5 ± 8.6	28.0 ± 18.0
Fasting insulin (IU/mL)	12.6 ± 7.2	26.0 ± 20.1*	18.8 ± 16.0*
HOMA-IR	2.8 ± 1.7	7.2 ± 5.7*	7.2 ± 7.6*
Adiponectin (ng/mL)	12597.2 ± 7237.6	7567.5 ± 4193.5*	7484.2 ± 4563.9*

*P value versus NGT group (significant at the 0.05 level).

**Table 2:** Correlations.

	Circulating adiponectin	
Age	*r*-value	0.295(**)
	*P* value	0.002
BMI	*r*-value	−0.008
	*P* value	0.931
AST	*r*-value	−0.279(**)
	*P* value	0.004
ALT	*r*-value	−0.348(**)
	*P* value	0.0001
HDL-cholesterol	*r*-value	0.418(**)
	*P* value	0.0001
LDL-cholesterol	*r*-value	0.128
	*P* value	0.206
Triglycerides	*r*-value	−0.453(**)
	*P* value	0.0001
Hs-CRP	*r*-value	−0.169
	*P* value	0.085
Fasting glucose	*r*-value	−0.375(**)
	*P* value	0.0001
HbA1C	*r*-value	−0.328(**)
	*P* value	0.001
Fasting insulin	*r*-value	−0.537(**)
	*P* value	0.0001
HOMA-IR	*r*-value	−0.623(**)
	*P* value	0.0001

**Correlation is significant at the 0.01 level (2-tailed).

**Table 3:** Clinical characteristics of study patients by CVRFs load.

Variables	Low-risk subjects *n* = 52	High-risk subjects *n* = 55	*P* value
Age	57.7 ± 9.1	55.2 ± 10.8	0.200
Sex (M/F)	13 (25%)	27 (49%)	0.01
BMI	31.8 ± 6.7	33.7 ± 6.2	0.135
CVRFs median (range)	2 (range 0–2)	3 (range 3–4)	0.002
Systolic blood pressure (mmHg)	128.1 ± 18.0	137.9 ± 18.2	0.006
Diastolic blood pressure (mmHg)	72.7 ± 12.5	77.7 ± 11.1	0.032
Mean arterial pressure (mmHg)	94.7 ± 14.2	99.0 ± 14.4	0.126
Fasting blood glucose (mg/dL)	93.7 ± 19.0	128.2 ± 45.8	0.0001
HBAIC (%)	5.7 ± 0.6	6.9 ± 1.8	0.0001
HDL-cholesterol (mg/dL)	56.5 ± 16.1	45.0 ± 15.4	0.0001
Triglycerides (mg/dL)	104.0 ± 47.5	186.0 ± 119.4	0.0001
Hs-CRP (mg/dL)	0.9 ± 0.4	0.9 ± 1.1	0.408
Fasting insulin (IU/mL)	11.9 ± 7.6	22.6 ± 17.4	0.0001
HOMA-IR	2.7 ± 1.8	7.0 ± 4.5	0.0001
AST (mg/dL)	22.4 ± 7.0	28.0 ± 14.3	0.011
ALT (mg/dL)	25.6 ± 12.1	32.6 ± 23.6	0.204
Adiponectin (ng/mL)	13701.1 ± 7051.5	6755.6 ± 3492.2	0.0001

**Table 4:** Correlations between adiponectin levels and number of CVRFs.

Correlations			CVRF's	Adiponectin
Spearman's rho	CVRF's	Correlation coefficient	1.000	−0.430(**)
		Sig. (2-tailed)		0.0001
		*N*	107	107
	Adiponectin	Correlation coefficient	−0.430(**)	1.000
		Sig. (2-tailed)	0.0001	
		*N*	107	107

**Correlation is significant at the 0.01 level (2-tailed).
